# Prolonged Intubation Induced Tracheoesophageal Fistula in Suspected Meningococcal Sepsis with ARDS: A Case Report

**DOI:** 10.31729/jnma.3890

**Published:** 2018-12-31

**Authors:** Ramesh Rana, Rikesh Sapkota, Binesh Shakya, Samir Gautam

**Affiliations:** 1 Department of Internal Medicine, Gautam Buddha Community Heart Hospital, Butwal, Nepal; 2 Department of Anaesthesiology, Gautam Buddha Community Heart Hospital, Butwal, Nepal; 3 Department of Cardiology, Gautam Buddha Community Heart Hospital, Butwal, Nepal

**Keywords:** *case report*, *endotracheal intubation*, *mechanical ventilation*, *tracheoesophageal fistula*

## Abstract

Tracheoesophageal fistula is an abnormal communication between trachea and esophagus. Benign acquired types are rare with the incidence of less than 1%. Prolonged endotracheal intubation remains the most common cause. We are reporting a 28 years old female patient presented with chief complaint of a cough after each meal intake in the outpatient clinic. She had a recent history of admission in the intensive care unit with prolonged intubation (11 days). Her general physical examination, laboratory examination, and chest x-ray were normal. Esophagogastroscopy was performed and revealed communication between upper esophagus and trachea approximately at 14–17cm embedded in longitudinal mucosal folds of the esophagus. She was referred to the higher center for surgical repair. Though, a rare complication, high suspicion is necessary to accurately diagnose the disease in a patient with the history of prolonged intubation.

## INTRODUCTION

Tracheoesophageal fistula (TEF) is an abnormal connection between trachea and esophagus. Acquired benign TEF is an uncommon complication; however, endotracheal intubation remains the most common cause with the incidence of <1%, first reported by D'Avignon (1956) and Mounier-Kuhn (1958).^1–4^

A cough after swallowing is the chief presentation of TEF with or without other associated comorbidities such as pneumonia. Bronchoscopy, esophagogastroscopy, and computed tomography are the investigations of choice. Early diagnosis and surgical correction have a better prognosis.^1,4,5^

We are reporting a rare case diagnosed early on esophagogastroscopy before the complication such as pneumonia.

## CASE REPORT

28 years old female patient referred to our center with chief complains of 7 days fever associated with generalized body rash and shortness of breath. The patient was intubated on day 3 of admission, a provisional diagnosis of meningococcal meningitis was made clinically, and lumbar puncture was not done (platelet counts: 21,000). Despite antibiotics therapy till day 7, there was persistent fever (max. recorded of 102.0°F); therefore, antitubercular therapy was started empirically. Endotracheal tube obstruction was noted on day 5 and day 7 of intubation; subsequent re-intubation was done twice. Patient was extubated on day 11, and fever subsided on day 14. Her post-extubation hospital stay was uneventful except an occasional cough during swallowing after removal of a nasogastric tube which we initially thought of post-extubation minor complication; eventually, she was discharged on day 18, with antitubercular therapy category I national regimen. During the first week of regular follow up, she presented to the outpatient department with a recurrent cough after each food intake. She had no associated symptoms of hoarseness of voice, dysphagia, fever, vomiting, difficulty in breathing, or chest pain.

**Figure 1.**
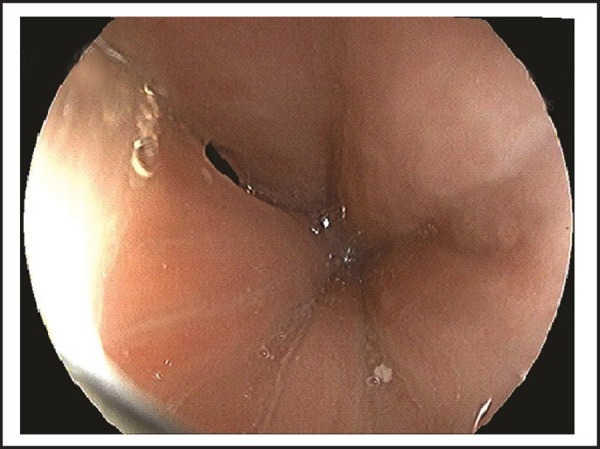
Partially visualized tracheoesophageal fistula embedded in longitudinal mucosal folds of oesophagus.

**Figure 2.**
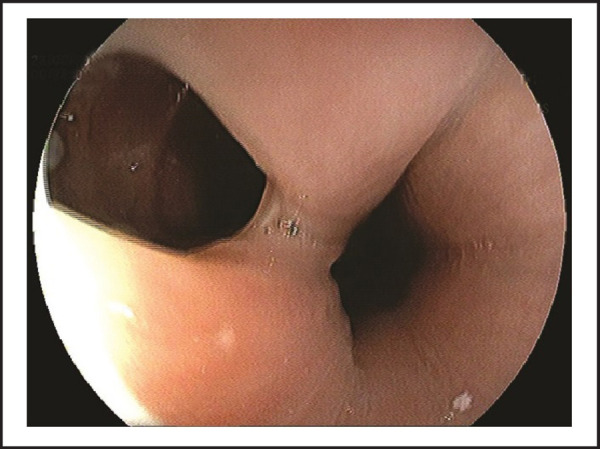
Demonstrating tracheoesophageal fistula with full air insufflations.

**Figure 3.**
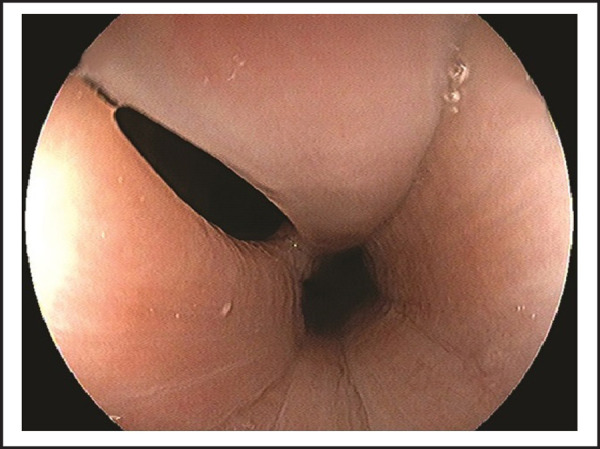
Highlighting whole dimensions of tracheoesophageal fistula with partially air insufflations.

On evaluation, she had normal vitals and physical examination. Her laboratory investigations were within normal limits and no abnormality detected on chest x-ray. We performed esophagogastroscopy, which revealed abnormal communication on upper esophagus with trachea (TEF) approximately at 14–17cm from upper incisor teeth, which was partially visualized at the beginning and hidden in collapsible longitudinal mucosal folds (Figure 1); further full air insufflations demonstrated an approximate length of 3 cm tracheoesophageal fistula (Figure 2 and Figure 3). She was managed conservatively to prevent further complications and then referred to the higher center for appropriate surgical correction of TEF.

## DISCUSSION

Benign acquired tracheoesophageal fistula is an extremely rare complication having multiple etiologies. Prolonged mechanical ventilation remains the prime cause, and if left untreated may lead to recurrent pulmonary infections leading to death.^6^ These cases commonly present with a cough after swallowing or with features of pneumonia. In case of high suspicions, bronchoscopy is the gold standard as it identifies the site of TEF in relation with anatomic location (glottis, cricoid cartilage, carina, and with a possible tracheal stoma), size of fistula, length of the segment. Whereas, esophagogastroscopy is the second investigation of choice; however, it has fewer chances to highlight the TEF especially when its dimension is reduced, and may be hidden by longitudinal mucosal folds. Computed tomography scan is another investigation which can confirm the presence of TEF, associated tracheal stenosis, and bronchopulmonary suppuration. A chest x-ray can be a supportive investigation to rule out associated co-morbidities such as pneumonia.^2,7,8^ Several therapeutic methods such as endoluminal stenting, fibrin glue application, and surgical correction have been described for post-intubation TEF; however, single stage operation approach is preferred choice over previous multi-stage operation.^4,7,9^

Prolonged ventilation induced TEF usually develops after 15–200 days. In our case, intubation duration was 11 days which was shorter than reported in the literature;^5,10^ however, multiple intubations due to tube blockade, restlessness, and use of steroid for suspected meningococcal/tubercular meningitis might be the predisposing factors for TEF early in our case. Copious productive cough intermittently and coughing after each food intake were the clinical presentations as in the literature. Bronchoscopy remains the investigation of choice; however, we performed esophagogastroscopy and were able to diagnose TEF in the proximal esophagus. Although, a rare complication, it could be seen in clinical practice in cases with prolonged ventilation. This was a good learning experience for us working daily in intensive care unit; furthermore, by selecting appropriate endotracheal tube cuff size, pressure monitoring, and avoiding hypermobility of tube and patient, we can minimize the risk of intubation-related complications like benign TEF.^4,5^

We successfully highlighted TEF early prior to serious complications. Our study has some limitations. As our hospital is located in rural area of the country, we could not perform bronchoscopy or CT scan as diagnostic modality and surgical correction/ repair of the case in our center due to unavailability of these facilities.

In conclusion, cough after swallowing in post-intubated cases is an alarming symptom of TEF; therefore, it should be ruled out in high suspicion cases. Further, those working in the intensive care unit should consider minimizing the incidence of TEF in prolonged intubated cases.

## References

[1] Bolca C, Päväloiu V, Fotache G, Dumitrescu M, Bobocea A, Alexe M (2017). Postintubation Tracheoesophageal Fistula-Diagnosis, Treatment and Prognosis. Chirurgia (Bucur)..

[2] Lee J-E, Chang M-Y, Kim KH, Jung YH. (2011). Post-intubation tracheoesophageal fistula with posterior glottic web. Clin Exp Otorhinolaryngol..

[3] Bibas BJ, Cardoso PFG, Minamoto H, Pêgo-Fernandes PM. (2018). Surgery for intrathoracic tracheoesophageal and bronchoesophageal fistula. Ann Transl Med..

[4] Paraschiv M. (2014). Tracheoesophageal fistula-a complication of prolonged tracheal intubation. J Med Life..

[5] Kucuk C, Arda K, Ata N, Turkkani MH, Yildiz ÖÖ (2016). Tracheomegaly and tracheosephagial fistula following mechanical ventilation: A case report and review of the literature. Respir Med Case Rep.

[6] Muniappan A, Wain JC, Wright CD, Donahue DM, Gaissert H, Lanuti M (2013). Surgical treatment of nonmalignant tracheoesophageal fistula: a thirty-five year experience. Ann Thorac Surg..

[7] Santosham R. (2018). Management of Acquired Benign Tracheoesophageal Fistulae. Thorac Surg Clin..

[8] Oe K, Araki T, Hayashi K, Yamagishi M. (2017). Tracheoesophageal fistula after long-term intubation. Intern Med..

[9] Puma F, Vannucci J, Santoprete S, Urbani M, Cagini L, Andolfi M (2017). Surgery and perioperative management for post-intubation tracheoesophageal fistula: case series analysis. J Thorac Dis..

[10] Rao SV, Boralkar AK, Jirvankar PS, Sonavani MV, Kaginalkar VR, Chinte C. (2016). Tracheoesophageal Fistula following Endotracheal Intubation for Organophosphorus Poisoning. J Assoc Physicians India..

